# Educated but on Social Security Disability Insurance: Minorities’ Diminished Returns

**DOI:** 10.31586/jbls.2024.1108

**Published:** 2024-11-09

**Authors:** Shervin Assari, Babak Najand, Hossein Zare, Amanda Sonnega

**Affiliations:** 1 Marginalized-Related Diminished Returns (MDRs) Research Center, Los Angeles, CA, USA; 2 Department of Family Medicine, Charles R. Drew University of Medicine and Science, Los Angeles, CA, USA; 3 Department of Urban Public Health, Charles R. Drew University of Medicine and Science, Los Angeles, CA, USA; 4 Department of Health Policy and Management, Johns Hopkins Bloomberg School of Public Health, Baltimore, MD, USA; 5 School of Business, University of Maryland Global Campus (UMGC), Adelphi, MD, USA; 6 Institute of Social Research, University of Michigan, Ann Arbor, MI, USA

**Keywords:** Social Security Disability Insurance, Educational Attainment, Minorities’ Diminished Returns, Racial Disparities, Latino, Black, Understanding America Study

## Abstract

**Background:**

Educational attainment is widely regarded as a key predictor of economic and social outcomes in later life, including the likelihood of receiving Social Security Disability Insurance (SSDI). According to the Minorities’ Diminished Returns (MDRs) theory, however, the benefits of education may be less pronounced for racial and ethnic minorities compared to non-Latino Whites. This study investigates whether the effects of education on the likelihood of receiving SSDI differ by race and ethnicity, focusing on Black and Latino Americans.

**Objective:**

The primary aim of this study was to examine the relationship between educational attainment (measured in years of schooling) and the likelihood of receiving SSDI, with a specific focus on exploring how this relationship varies by race and ethnicity, in line with the MDRs framework.

**Methods:**

Data were drawn from the Understanding America Study (UAS), a nationally representative, internet-based panel survey. The sample included Black, Latino, and non-Latino White U.S. adults. Our sample size was 12,975 adults over the age of 18. Logistic regression models were used to assess the association between educational attainment and receiving SSDI, adjusting for demographic variables such as age, sex, employment status, and marital status. Interaction terms between race/ethnicity and educational attainment were included to explore whether the returns on education varied across racial and ethnic groups.

**Results:**

Higher educational attainment was significantly associated with a lower likelihood of receiving SSDI in the overall sample. However, consistent with the MDRs framework, the protective effect of education was significantly weaker for both Black and Latino individuals compared to non-Latino Whites. Black and Latino participants with similar levels of education as their non-Latino White counterparts were more likely to receive SSDI, reflecting diminished returns on educational attainment for these groups.

**Conclusion:**

This study provides strong evidence supporting the MDRs theory, demonstrating that the protective effects of education on the likelihood of receiving SSDI are not equally distributed across racial and ethnic groups. Black and Latino Americans experience weaker returns on their education when it comes to avoiding SSDI, likely due to structural inequalities and systemic barriers. These findings highlight the need for policies that address not only educational disparities but also the broader societal factors that limit the benefits of education for racial and ethnic minorities.

## 1. Introduction

Social Security Disability Insurance (SSDI) serves as a vital safety net for individuals who are unable to work due to significant health issues or disabilities [1]. Established in 1956, SSDI was designed to provide financial support to those who have paid into the Social Security system but are now physically or mentally incapable of maintaining gainful employment [2, 3]. This program offers crucial financial assistance to individuals who are otherwise at risk of falling into poverty due to their inability to participate in the labor force [4]. However, high rates of SSDI utilization can also serve as an indicator of broader public health and social challenges, highlighting the need for preventative measures to reduce the burden of disability in society [5, 6].

In a healthy society, where healthcare access, education, and employment opportunities are equitable and robust, the need for SSDI should, in theory, be lower. Improved public health measures, early intervention programs, and access to healthcare can help prevent or delay the onset of disabling conditions [7]. Similarly, safer workplaces, inclusive employment practices, and robust rehabilitation services could allow individuals with health conditions or disabilities to remain active in the workforce [8–10]. High rates of SSDI utilization, on the other hand, may signal gaps in the healthcare system, occupational safety, or social support structures that fail to address the needs of individuals before they reach a point of permanent disability [11, 12].

Moreover, the disproportionate reliance on SSDI among certain populations, such as racial and ethnic minorities, can be attributed in part to lower economic stability, reduced savings, and diminished wealth and health compared to their non-Latino White counterparts [13–16]. These disparities are rooted in deeper structural and historical inequalities within society. Segregation, Jim Crow laws, and the unequal exposure of racial and ethnic groups to environmental toxins and chronic stress are key drivers of the disparities in health and wealth [17]. Factors such as limited access to quality healthcare, more hazardous working conditions, and fewer opportunities for upward mobility further contribute to higher rates of disability in these communities. Addressing these inequities through comprehensive public health interventions and structural reforms would not only reduce the reliance on SSDI but also foster a healthier, more equitable society, where individuals are less likely to face disabling conditions that hinder their ability to work [4].

Higher education is one of the key factors that can reduce the need for SSDI by promoting better health, greater employment stability, and improved access to resources [18]. Individuals with higher levels of education are more likely to secure jobs that offer safer working conditions, health benefits, and opportunities for professional development, all of which contribute to better long-term health outcomes [19–23]. Additionally, education often provides people with the knowledge and skills needed to make healthier lifestyle choices, access preventive healthcare, and manage chronic conditions more effectively, reducing the likelihood of developing disabilities that impair their ability to work [24–26]. Furthermore, higher education increases financial stability and job security, which in turn can act as a buffer against the need for SSDI [27–30]. Well-paying jobs with benefits are more likely to offer accommodations for employees who face health challenges, allowing them to remain in the workforce longer [29, 31–36]. Conversely, individuals with lower levels of education often have less access to such opportunities, working in jobs with fewer protections and more physically demanding or hazardous conditions. These factors increase the risk of disability and subsequent reliance on SSDI. By promoting access to education and addressing educational disparities, society can reduce the overall need for SSDI, improving both individual well-being and public health outcomes.

While individuals with higher levels of education tend to be healthier, work longer, and have better financial stability, recent research highlights that the effects of education on these conditions are not equally experienced across racial and ethnic groups [37–41]. The *Minorities’ Diminished Returns (MDRs)* theory [42] posits that the health benefits of education, as well as other SES indicators, are weaker for racial and ethnic minorities compared to White populations. For example, while higher levels of education are associated with improved health for most individuals, Black and Latino individuals often experience diminished returns from their educational attainment [37–43]. Similarly, highly educated Black and Latino people may work in worse occupations, earn less income, and accumulate less wealth [44]. These diminished returns may manifest in higher stress levels, less secure employment opportunities, and less access to high-quality healthcare, despite equivalent levels of education [42]. Structural racism, discrimination, and unequal access to resources are to blame for the weaker advantages of educational attainment for marginalized population [42].

This study builds on the MDRs framework [42] by examining the association between years of schooling and receipt of SSDI across different racial and ethnic groups, focusing on Non-Latino White, Non-Latino Black, and Latino adults. Specifically, we aim to explore whether the protective effect of education on the receipt of SSDI is weaker for Black and Latino adults compared to White adults, after controlling for key sociodemographic factors such as age, sex, employment status, and marital status.

By focusing on racial and ethnic variation in the effects of education on receipt of SSDI, this study contributes to an emerging literature highlighting the inequitable distribution of economic and health benefits of SES resources in the United States [37, 45]. Understanding these diminished returns is critical for informing public health interventions and policies aimed at reducing economic and racial and ethnic disparities and promoting economic and health equity for all racial and ethnic groups.

## 2. Methods

The Understanding America Study (UAS) [46–50] is a large, nationally representative, internet-based survey conducted by the University of Southern California (USC). The UAS is designed to gather extensive insights on a wide array of social, economic, and health-related issues across the U.S. population. The study employs probability-based sampling methods, drawing from post-office delivery sequence files to recruit participants. To ensure full inclusivity and representativeness, individuals who do not have internet access are provided with internet-enabled devices, such as tablets, along with internet services, enabling them to participate in the surveys.

The UAS collects detailed data on numerous domains, including well-being, retirement readiness, cognitive functioning, health behaviors, and personality traits. These surveys are administered regularly—either annually or biennially—to capture longitudinal data on participants. In addition to socioeconomic and behavioral variables, UAS includes health-related metrics to provide continuous assessments of the participants’ health status over time.

Our analytical sample was 12,975 in this analysis, selected based on available data on SSDI, being Latino or non-Latino, and Black or White. Participants could be immigrant.

The key independent variable in this study is educational attainment, measured as years of schooling. Education is self-reported by participants, capturing the total number of years of formal education completed. This continuous measurement of education enables us to capture the gradient, rather than a threshold effect, of declining SSDI receipt associated with each additional year of schooling. This approach aligns with previous research showing that each added year of education is a significant predictor of both economic and health outcomes [42]. Consequently, our effect size will reflect how each incremental year of education influences the need for SSDI, both overall and across different racial and ethnic groups.

Several covariates were included in the analysis to account for potential confounders. Age was measured in years, and sex was self-reported as male or female. Employment status at the time of the survey was categorized as employed or not employed, providing insight into the participants’ current work situation, which is an important determinant of health. Marital status was also self-reported and dichotomized as married or not married, given that marital status has been shown to influence health outcomes through social support and economic stability. These variables were included in the models to isolate the independent effect of education on SSDI, while accounting for other demographic and socioeconomic factors. Nativity was US-born and non-US non (immigrant).

For this study, we used data from the first wave of the UAS (year 2014). The primary outcome variable was SSDI receipt, measured using a binary variable (1 = received, 0 = not received). In addition to their SSDI receipt, participants provided demographic information, including their sex, age, marital status, country of birth (U.S.-born vs. non-U.S.-born), and current employment status. These variables were included as covariates to account for potential confounding effects on retirement preparedness.

The UAS [46–50] offers a robust dataset for investigating the relationship between socioeconomic indicators and social security outcome as well as their variations across racial and ethnic groups. Its extensive data collection and diverse sample allow for an in-depth exploration of how race and ethnicity may moderate the associations between social determinants and economic and social security outcomes.

### 2.1. Data Analysis

First, we described our sample in the pooled sample. Next, logistic regression models were employed to examine the relationship between educational attainment (measured by years of schooling) and SSDI (0 vs. 1), while adjusting for key demographic factors such as age, gender, employment status, marital status, and nativity. Race and ethnicity were the moderators. Two models were specified for the analysis: Model 1 included educational attainment, race, ethnicity, and /other control variables, without interaction terms. Model 2 incorporated interaction terms between race/ethnicity and years of education to assess whether the relationship between educational attainment and retirement preparedness varied by race and ethnicity. This analytical approach allowed for a detailed assessment of potential differences in how educational attainment impacts SSDI receipt across racial and ethnic groups, with a particular focus on the Minorities’ Diminished Returns (MDRs) theory [51–59]. According to MDRs, the effects of educational attainment on outcomes like SSDI may be less substantial for Latino and Black individuals compared to non-Latino Whites [42, 43, 60–64]. Results were reported as odds ratio (OR), along with p-values and 95% confidence intervals (CIs).

### 2.2. Ethical Considerations

All participants had previously consented to their involvement in UAS-related research as part of their enrollment in the UAS panel. For this specific analysis, the University of Southern California’s Institutional Review Board (IRB) required additional informed consent procedures. Participants were explicitly informed that individuals with significant cognitive impairments, who may not fully understand their rights, were excluded from the study. To confirm participants’ understanding of their rights, they were asked to complete a brief multiple-choice quiz before giving their final consent. The study received full approval from the USC IRB.

## 3. Results

Overall, 12,975 participants entered our analysis. Table 1 provides descriptive data for the overall sample. The average age of participants was 47.34 years (SD = 16.23), and the mean years of education was 11.24 (SD = 2.28). The majority of the sample identified as White (87.0%), with Black individuals comprising 13.0% of the sample. In terms of ethnicity, 84.4% of participants were non-Latino, while 15.6% were Latino. Most participants were U.S.-born (91.5%), with 8.5% identifying as immigrants. The sample had more women (60.4%) than men (39.6%). Marital status was evenly distributed, with 50.2% of participants being married and 47.6% not married. More than half of the sample (56.7%) reported being employed, while 41.1% were not working. Finally, 6.2% of participants reported receiving Social Security Disability Insurance (SSDI), while 93.8% did not.

As shown in Table 2, higher educational attainment was associated with lower odds of receiving SSDI; specifically, each additional year of schooling decreased the likelihood of SSDI receipt (OR = 0.823, 95% CI: 0.796, 0.852, p < 0.001). Black individuals had higher odds of receiving SSDI compared to White individuals (OR = 1.297, 95% CI: 1.060, 1.588, p = 0.012), while Latino individuals were less likely to receive SSDI compared to non-Latino individuals (OR = 0.491, 95% CI: 0.366, 0.659, p < 0.001). Immigrants also had lower odds of receiving SSDI compared to U.S.-born individuals (OR = 0.553, 95% CI: 0.368, 0.832, p = 0.005). Age was positively associated with SSDI receipt, with each additional year of age slightly increasing the odds (OR = 1.009, 95% CI: 1.005, 1.014, p < 0.001). Women had lower odds of receiving SSDI compared to men (OR = 0.853, 95% CI: 0.728, 1.000, p = 0.049). Employment status showed a strong inverse association with SSDI receipt, as employed individuals had substantially lower odds (OR = 0.061, 95% CI: 0.046, 0.081, p < 0.001). Lastly, married individuals had higher odds of receiving SSDI compared to those who were unmarried (OR = 1.949, 95% CI: 1.649, 2.303, p < 0.001).

As shown in Table 3, educational attainment, measured in years of schooling, was negatively associated with SSDI receipt, indicating that more years of schooling reduced the odds of receiving SSDI (OR = 0.661, 95% CI: 0.591, 0.740, p < 0.001). However, interaction effects revealed that the protective impact of education on SSDI receipt varied by race and ethnicity. Specifically, the interaction between years of schooling and Black race was positively associated with SSDI receipt, suggesting that the protective effect of education was weaker for Black individuals compared to White individuals (OR = 1.186, 95% CI: 1.085, 1.296, p < 0.001). Similarly, the interaction between years of schooling and Latino ethnicity indicated that higher education had a reduced impact on lowering SSDI receipt for Latino individuals compared to non-Latino individuals (OR = 1.188, 95% CI: 1.050, 1.345, p = 0.006). The interaction between years of schooling and immigrant status was not significant (OR = 1.006, 95% CI: 0.872, 1.160, p = 0.936).

Age was positively associated with SSDI receipt, with each additional year of age slightly increasing the odds (OR = 1.010, 95% CI: 1.005, 1.014, p < 0.001). Women had lower odds of receiving SSDI than men (OR = 0.837, 95% CI: 0.713, 0.981, p = 0.028). Employment status showed a strong inverse association with SSDI receipt (OR = 0.060, 95% CI: 0.045, 0.080, p < 0.001), and married individuals had higher odds of SSDI receipt compared to those who were not married (OR = 1.932, 95% CI: 1.635, 2.284, p < 0.001).

## 4. Discussion

The primary aim of this study was to examine the relationship between educational attainment and the likelihood of receiving SSDI in the U.S. population. Specifically, we investigated whether this relationship varied by race and ethnicity within the framework of Minorities’ Diminished Returns (MDRs). According to MDRs theory, the health and economic benefits of education and other socioeconomic factors tend to be less substantial for racial and ethnic minorities than for non-Latino Whites. By analyzing these associations among Black, Latino, and non-Latino White individuals, we aimed to understand how disparities in SSDI receipt may be influenced by differential returns on education across these groups.

Our findings show that higher educational attainment is associated with a significantly lower likelihood of receiving SSDI, confirming the important role that education plays in promoting economic stability and reducing the need for disability insurance [19–23]. However, consistent with the MDRs framework [42], the protective effects of education on SSDI receipt are significantly weaker for Black and Latino individuals compared to non-Latino Whites. Even with comparable levels of education, Black and Latino participants are more likely to receive SSDI, reflecting the diminished returns of education for these groups. This suggests that higher education, while beneficial, does not provide the same degree of protection against disability-related financial need for racial and ethnic minorities as it does for non-Latino Whites.

These findings strongly support the Minorities’ Diminished Returns theory [42]. While education is a major social determinant of economic and health outcomes, the benefits of higher education are not distributed equally across racial and ethnic groups. For Black and Latino individuals, education yields less substantial reductions in the likelihood of SSDI receipt, despite similar educational achievements. Minorities’ Diminished Returns (MDRs) [41, 52, 57, 58, 61, 65–81] refer to the phenomenon where racial and ethnic minorities, particularly Black and Latino individuals, receive fewer benefits from socioeconomic resources—such as education—compared to their non-Latino White counterparts.

This diminished return on education underscores the structural inequities present in the U.S., where systemic barriers prevent racial and ethnic minorities from fully realizing the benefits of education. These disparities likely contribute to the increased reliance on SSDI among minority groups, even at higher educational levels. MDRs exist largely because of structural inequalities, systemic discrimination, and historical marginalization. For example, Black and Latino individuals may face labor market discrimination, limiting their ability to access high-quality jobs, even with comparable educational qualifications. Additionally, these groups often attend lower-quality schools and face greater challenges in accumulating wealth, which can lead to poorer health outcomes and increased susceptibility to disability. As a result, the protective effects of education are eroded by the societal barriers that prevent racial and ethnic minorities from fully translating educational attainment into economic security and health [82].

### 4.1. Implications

The findings from this study have several important implications for policy and practice. First, they highlight the need for policies that not only promote educational attainment but also address the structural barriers that limit the returns on education for racial and ethnic minorities. Improving access to high-quality education, reducing labor market discrimination, and creating more equitable job opportunities are essential steps toward reducing disparities in SSDI receipt. Furthermore, targeted public health and social programs aimed at reducing the health disparities that disproportionately affect Black and Latino communities are necessary to mitigate the underlying causes of higher disability rates among these groups. Addressing these broader societal factors is critical for ensuring that all individuals, regardless of race or ethnicity, can fully benefit from their educational achievements.

To address the diminished returns of education for racial and ethnic minorities, it is essential to tackle a range of systemic and structural factors. One of the primary issues is the unequal distribution of resources in public education. Schools in minority communities often receive less funding due to property tax-based funding models, resulting in lower-quality education for Black and Latino students. Additionally, labor market discrimination continues to disadvantage minorities, limiting their access to well-paying, stable jobs with health benefits and disability accommodations. These structural barriers not only diminish the financial benefits of education for minorities but also increase their exposure to hazardous work conditions, contributing to higher disability rates and greater reliance on SSDI. Addressing these diminished returns will require concerted efforts to promote social justice and reduce social stratification and racism in the U.S.

### 4.2. Limitations

This study has several limitations that should be considered when interpreting the results. First, the data used in this analysis were cross-sectional, which limits causal conclusions about the relationship between education and SSDI receipt. Longitudinal data would provide a more comprehensive understanding of how educational attainment affects disability risk over time. Second, while we controlled for several key demographic factors, other potential confounders, such as early-life socioeconomic conditions, health conditions, access to healthcare, and occupational hazards, were not included in the analysis. Finally, the self-reported nature of SSDI receipt may introduce reporting bias. Despite these limitations, this study expands existing knowledge on the social determinants of SSDI in the U.S.

## 5. Conclusion

In summary, this study provides additional evidence that although educational attainment is a key factor in reducing the likelihood of SSDI receipt, its benefits are not equally distributed across racial and ethnic groups. Black and Latino individuals experience diminished returns on their education when it comes to avoiding disability and relying on SSDI, likely due to structural inequalities and systemic barriers. These findings underscore the need for policies that not only promote educational attainment but also address the broader societal factors that limit the ability of racial and ethnic minorities to fully benefit from their education. Reducing disparities in SSDI receipt requires a multifaceted approach that tackles both the educational and structural inequalities faced by minority communities.

## Figures and Tables

**Table 1.** Descriptive Data Overall (n = 12,975)

	Mean	Std. Deviation
Age	47.34	16.230
Educational Attainment (Years)	11.24	2.280
	n	%
Race		
White	11287	87.0
Black	1688	13.0
Ethnicity		
Non-Latino	10949	84.4
Latino	2026	15.6
Immigrant		
No	11869	91.5
Yes	1106	8.5
Gender		
Men	5141	39.6
Women	7832	60.4
Married		
No	6178	47.6
Yes	6513	50.2
Working		
No	5333	41.1
Yes	7354	56.7
SSDI Receipt		
No	12165	93.8
Yes	810	6.2

Source: Understanding America Study (UAS), SSDI: Social Security Disability Insurance

**Table 2.** Summary of Logistic Regression without Interaction

	B	S.E.	Sig.	Exp(B)	95% C.I.for EXP(B)	
					Lower	Upper
Ethnicity (Latino)	−.711	.150	< .001	.491	.366	.659
Race (Black)	.260	.103	.012	1.297	1.060	1.588
Nativity (Immigrant)	−.592	.209	.005	.553	.368	.832
Age (Year)	.009	.002	< .001	1.009	1.005	1.014
Gender (Female)	−.159	.081	.049	.853	.728	1.000
Employment (Working)	−2.801	.146	< .001	.061	.046	.081
Marital Status (Married)	.667	.085	< .001	1.949	1.649	2.303
Years of Schooling	−.194	.017	< .001	.823	.796	.852

Source: Understanding America Study (UAS), Outcome: Social Security Disability Insurance (SSDI) Receipt

**Table 3.** Summary of Logistic Regression without Interaction

	B	S.E.	Sig.	Exp(B)	95% C.I.for EXP(B)	
					Lower	Upper
Ethnicity (Latino)	−2.357	.643	< .001	.095	.027	.334
Race (Black)	−1.440	.469	.002	.237	.095	.594
Nativity (Immigrant)	−.576	.775	.457	.562	.123	2.566
Age (Year)	.010	.002	< .001	1.010	1.005	1.014
Gender (Female)	−.178	.081	.028	.837	.713	.981
Employment (Working)	−2.812	.146	< .001	.060	.045	.080
Marital Status (Married)	.659	.085	< .001	1.932	1.635	2.284
Years of Schooling	−.414	.057	< .001	.661	.591	.740
Years of Schooling × Immigrant	.006	.073	.936	1.006	.872	1.160
Years of Schooling × Black	.170	.045	< .001	1.186	1.085	1.296
Years of Schooling × Latino	.172	.063	.006	1.188	1.050	1.345

Source: Understanding America Study (UAS), Outcome: Social Security Disability Insurance (SSDI) Receipt
